# Numerical Study of Optical Nonreciprocal Transmission via Liquid Metamaterial Nonlinearity

**DOI:** 10.3390/ma18102241

**Published:** 2025-05-12

**Authors:** Tiesheng Wu, Xin Cheng, Yujing Lan, Zhenyu Li, Changpeng Feng, Yingshuang Huang, Yingtao Tang, Hongyun Li, Yiwei Peng

**Affiliations:** 1 Guangxi Key Laboratory of Wireless Broadband Communication and Signal Processing, School of Information and Communication, Guilin University of Electronic Technology, Guilin 541004, China; tieshengw@guet.edu.cn (T.W.); h_yingshuang@163.com (Y.H.); a836677801@163.com (Y.T.);; 2 The Electronic Fifth Institute of the Ministry of Industry and Information Technology, Guangzhou 511370, China; pyw0120@163.com

**Keywords:** nonreciprocal metasurface, liquid metamaterials (LMMs#), optical nonlinearity, optical isolation

## Abstract

This study proposes and numerically demonstrates a novel nonreciprocal electromagnetic metasurface by integrating a highly nonlinear liquid metamaterial (LMM) with a simple two-dimensional silicon dielectric grating. The transmission characteristics of the proposed structure were investigated using a full-vector finite-element method. We demonstrated that the proposed subwavelength-thickness metasurface achieves a transmission coefficient contrast of up to 0.96 between forward and backward propagation. Highly nonlinear LMMs, when employed as nonreciprocal media, significantly lower the radiation power needed to induce a nonlinear response compared to natural materials. Furthermore, we numerically analyzed the effects of the grating’s structural parameters, LMM thickness, and packing fraction on transmittance. The proposed design holds promise for applications in optical isolators.

## 1. Introduction

Metasurfaces have emerged as a prominent research area in recent years, experiencing rapid advancements in their theoretical design and industrial fabrication [1,2]. Electromagnetic metasurfaces typically consist of an array of resonant subwavelength elements arranged in specific patterns. Through the precise design of these elements and their spatial arrangement, metasurfaces can be engineered to perform various functions, including light focusing, beam steering, holography, and chiral electromagnetic wave (EW) polarization control [3,4,5,6]. Consequently, metasurfaces remain a key focus of research in academia and industry [7,8,9], offering broad applications in optics, communications, and sensing. In optical communication systems, metasurfaces can enable the fabrication of critical components, such as optical isolators and circulators. Notably, these components function as optical nonreciprocal devices [10,11,12,13,14], and the development of miniaturized, high-performance, nonreciprocal optical elements remains a critical challenge in modern communication systems. Nonreciprocity describes the directional dependence of a system on an input signal. Specifically, the system exhibits asymmetry under time-reversal transformation, and achieving optical nonreciprocity requires breaking the Lorentz reciprocity, which significantly constrains the possible implementation methods. Three materials are commonly used to achieve nonreciprocity: magneto-optical, time-varying, and nonlinear materials. Traditional optical nonreciprocal devices rely on the magneto-optical Faraday effect to break time-reversal symmetry, and they are typically bulky [15,16,17,18]. With advances in optical systems and devices, miniaturized nonreciprocal systems have become increasingly important in optical communications, signal processing, and related fields. Lei et al. proposed a superconducting ceramic superstructured photonic crystal (SCMPC) to analyze the transmission characteristics of electron beams [19]. Their results indicated that, within the 696–715 THz band, the periodic SCMPC enabled asymmetric absorption–transmission of electron waves and exhibited double-band absorption. In subsequent research, Lei et al. proposed a plasma metastructure–photonic crystal system to achieve asymmetric transmission in a dual-channel configuration [20]. Owing to asymmetry in plasma’s spatial structure distribution and the magneto-optical effect of light, the propagation properties of EWs differ significantly when incident from the forward or backward directions across various wavelength bands. Their findings showed that the structure enabled backward transmission within an operating bandwidth of 2.15–2.85 GHz and forward transmission within 7.07–7.67 GHz. These studies achieved dual-band and high-bandwidth nonreciprocal transmission, paving the way for further advancements in nonreciprocal device research.

Asymmetric transmission devices based on optical nonlinearity have long been a subject of research interest. Optical devices integrating nonlinear materials with metasurfaces enable all-optical control without requiring an external magnetic field, while maintaining a compact size and high isolation [21,22,23,24,25]. However, natural materials typically exhibit low optical nonlinearity and require extremely high light intensities to achieve the desired effect. To enhance performance, researchers have commonly substituted natural materials with artificial metamaterials [26,27,28]. Liquid metamaterials (LMMs) are synthetic materials exhibiting unique and highly tunable mechanical, optical, and electromagnetic properties [29,30,31,32,33,34]. Metamaterials exhibit properties that are absent in natural materials, owing to their nanoscale structural design rather than the properties of their constituent components [35]. Liquid metamaterials are metamaterials of supramolecules whose optical response depends on the contribution of liquids. This dependence stems from coupling with the liquid background of the supramolecules or resonance within the liquid-structured supramolecules. The liquid metamaterial can be divided into the liquid background metamaterial and the liquid-cell metamaterial. The liquid background metamaterial consists of metamaterial in water solvent, metamaterial in LC, and nanoparticles in liquid. The liquid-cell metamaterial consists of water-cell metamaterial and liquid-metal-cell metamaterial [36]. LMMs are highly adjustable and reconfigurable, making them attractive for research [37,38]. Despite extensive research on LMMs, few studies have explored their integration with the asymmetric propagation properties of electron beams.

This study proposes and investigates a two-dimensional (2D) metasurface-based nonreciprocal optical device integrating a nonlinear LMM with a silicon grating structure. The LMM used in this study was a colloidal suspension prepared by doping gold nanoparticles in water-based materials. Its nonlinear response is derived from the combined effect of the nonlinearity of the liquid materials and metal nanoparticles. Due to local field enhancement and resonant absorption, metal nanoparticles exhibit obvious third-order optical nonlinearity, and this makes the nonlinearity coefficient of LMMs much higher than that of natural materials. The nonreciprocal transmission function of the metasurface-based design was achieved through the nonlinearity of the LMM, to break the Rayleigh–Carson reciprocity theorem. Simulations indicated that the proposed structure exhibited significant nonreciprocity at an incident light intensity of 18 kW/cm^2^ and operating wavelength of 1549.2 nm. Under these conditions, the forward transmittance was 0.004, whereas the reverse transmittance reached 0.964, yielding a transmittance contrast of 0.96. The proposed metasurface design offers several advantages. First, it facilitates strong optical nonreciprocity within an ultrathin layer. Specifically, it achieves strong optical nonreciprocity in a layer thinner than 1 µm, which is smaller than the wavelength of the incident radiation. Second, despite its small thickness, the metasurface exhibits a large transmission gap in different directions, ensuring effective optical isolation. Finally, by tuning its structural parameters, the metasurface can provide optical isolation at a specified frequency, enhancing its adaptability.

## 2. Design and Simulation Methods

**Figure 1:**
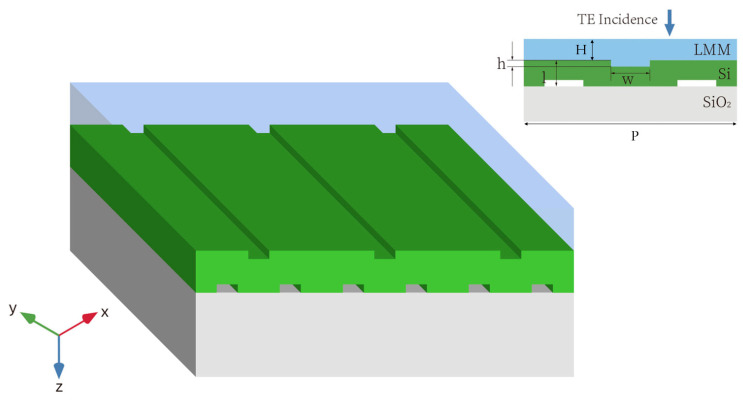
Structural two-dimensional and three-dimensional view of the metasurface.

Figure 1 shows a schematic of the proposed structure. Silicon (n = 3.4) was selected as the waveguide layer material, with a thickness of l = 181 nm, while silicon dioxide (n = 1.46) served as the semi-infinite substrate. The substrate thickness has a negligible effect on the optical spectra when it exceeds 500 nm, as the local field resonance primarily occurs within the silicon waveguide. Therefore, a semi-infinite substrate was used instead of a finite-thickness “thick” substrate, so as to simplify the simulations. Periodic rectangular grooves were etched into the silicon surface, with a grating period of P = 1192 nm, groove width w = 80 nm, and groove depth h = 10 nm. The lower surface grooves were filled with air, while the upper surface was coated with an LMM layer of thickness H = 200 nm. This study focuses on transverse electric polarization, where the electric field oscillates along the y-direction.

Nonreciprocity is a phenomenon wherein an EW propagating in opposite directions within a material experiences different electromagnetic losses, phase shifts, and other characteristics. The fundamental principle involves utilizing light–matter interactions to break time-reversal symmetry. To achieve nonreciprocity, it is essential to first understand the conditions under which reciprocity occurs. Consider sources $J_{1}^{e}$, $J_{1}^{m}$ generating fields $E_{1}$, $H_{1}$, and sources $\;J_{2}^{e}$, $J_{2}^{m}$ generating fields $E_{2}$, $H_{2}$:

(1) $$\nabla \times E_{1}=i\omega \mu H_{1}-J_{1}^{m},\nabla \times E_{2}=i\omega \mu H_{2}-J_{2}^{m}$$

Multiplying both sides of Equation (1) by $H_{1}$ or $H_{2}$ and subtracting, we obtain the following result under the condition $\mu =\mu^{T}$:

(2) $$i\omega H_{2}\mu H_{1}-i\omega H_{1}\mu H_{2}=i\omega H_{1}\mu^{T}H_{2}-i\omega H_{1}\mu H_{2}=0$$

Combining Equations (1) and (2), we derive

(3) $$H_{2}\cdot \nabla \times E_{1}-H_{1}\cdot \nabla \times E_{2}=H_{1}J_{2}^{m}-H_{2}J_{1}^{m}$$

Similarly, assuming $\epsilon =\epsilon^{T}$, we combine Equations (1) and (3) to derive

(4) $$\int_{V}E_{1}J_{2}^{e}-H_{1}J_{2}^{m}=\int_{V}E_{2}J_{1}^{e}-H_{2}J_{1}^{m}$$

If there are only electric field sources $J_{1}^{e}$ and $J_{2}^{e}$ in the area, Equation (4) can be simplified as $\int_{V}{[E}_{1}J_{2}^{e}-E_{2}J_{1}^{e}]dV=0$; this equation is called the Rayleigh–Carson reciprocity theorem. Rayleigh–Carson reciprocity holds under two conditions: $\mu =\mu^{T}$ and $\epsilon =\epsilon^{T}$. To achieve nonreciprocity, at least one of these conditions must be violated. The dielectric constant, also known as the relative permittivity or relative dielectric constant, is a physical quantity that describes a material’s response to an electric field. It indicates the material’s ability to store and conduct charges under the influence of an electric field. The dielectric constant of nonlinear materials depends on the electric field strength, and a metasurface with spatial asymmetry alters the electric field distribution, leading to different wave propagation characteristics in opposite directions. By combining the design of the metasurface with nonlinear materials, the nonreciprocity condition $\epsilon \neq \epsilon^{T}$ can be realized.

The electromagnetic response of a metasurface can be understood within the framework of a simple model. First, we considered a purely linear problem involving the interaction of a dielectric metasurface with an EW. For a typical plane EW incident on the metasurface, the electromagnetic response is primarily determined by the electric and magnetic dipole moments induced in the unit cells. The electric dipole moment is a system composed of two equal but opposite charges, usually expressed as the product of the charges and the distance between them, with the direction from the negative charge to the positive charge. The magnetic dipole moment is composed of a current loop or two approximately opposite magnetic charges, and it is usually related to the direction of the current and the area of the loop. The properties of these two dipole moments can be described by their dipole moments. The electric dipole moment is related to the charge distribution, while the magnetic dipole moment is related to the current distribution. For a general linear meta-atom, the relationship between the induced electric and magnetic dipole moments and external fields at the position of the meta-atom is given as follows [39]:

(5) $$p={\hat{\alpha }}_{ee}E+{\hat{\alpha }}_{em}H,m={\hat{\alpha }}_{me}E+{\hat{\alpha }}_{mm}H$$

where ${\hat{\alpha }}_{ee}$, ${\hat{\alpha }}_{mm}$, ${\hat{\alpha }}_{em}$, and ${\hat{\alpha }}_{me}$ represent the electric, magnetic, electromagnetic, and magnetoelectric polarizability dyadics (or tensors) of the inclusion, respectively. The polarization response of atoms and molecules in natural nonmagnetic materials is predominantly determined by electric polarizability ${\hat{\alpha }}_{ee}$. Due to the electrically small size of atoms (a<<λ), the magnetoelectric (${\hat{\alpha }}_{em}$, ${\hat{\alpha }}_{me}$) and magnetic (${\hat{\alpha }}_{mm}$) polarizabilities are negligible, as weak spatial dispersion effects of the a/λ and (a/λ)2 orders. Considering the relationship between the electric and magnetic fields in a plane wave, and assuming X-polarization, the dipole moment can be expressed as follows:

(6) $$p_{x}=\left(\alpha_{ee}^{xx}\pm Z_{0}^{-1}\alpha_{em}^{xy}\right)E_{x};m_{y}=\left(\pm Z_{0}^{-1}\alpha_{mm}^{yy}-\alpha_{em}^{xy}\right)E_{x}$$

where ± denotes the forward (along the *z*-axis) and backward (opposite to the *z*-axis) propagation directions of the plane wave, and Z_0_ represents the impedance of the surrounding medium. In Equation (6), we account for the reciprocity of the medium in the linear regime, which gives $\alpha_{me}^{yx}=-\alpha_{em}^{xy}$ [38]. Finally, the absolute values of the dipole moments are given by

(7) $${\left|p_{x}\right|}^{2}=\left({\left|\alpha_{ee}^{xx}\right|}^{2}+{\left|Z_{0}^{-1}\alpha_{em}^{xy}\right|}^{2}\pm 2Z_{0}^{-1}Re\left(\alpha_{ee}^{xx}\alpha_{em}^{xy*}\right)\right){\left|E_{x}\right|}^{2}$$

(8) $${\left|m_{y}\right|}^{2}=\left({\left|Z_{0}^{-1}\alpha_{mm}^{yy}\right|}^{2}+{\left|\alpha_{em}^{xy}\right|}^{2}\mp 2Z_{0}^{-1}Re\left(\alpha_{mm}^{yy}\alpha_{em}^{xy*}\right)\right){\left|E_{x}\right|}^{2}$$

where the asterisk corresponds to the complex conjugation. The grating asymmetry concerning the wave’s propagation direction induces spatial dispersion, leading to magnetoelectric coupling [24]. Therefore, the dipole-mode excitation is asymmetric regarding the light’s propagation direction. In particular, the near-field distribution produced by an electric dipole moment can be expressed as $E\left(r\right)=\left(3\left(p\cdot r\right)r-r^{2}p\right)r^{-5}$, where r represents the vector connecting the dipole to the observation point, and r represents the absolute value of the vector r. The near-field amplitude is proportional to the absolute value of the electric dipole moment. Asymmetric dipole excitation results in different near-field distributions for forward and backward illumination. Consequently, a significant difference in the amplitudes of electric dipoles excited by an EW propagating forward or backward leads to a significant difference in the electric field amplitudes near the dipoles. In nonlinear optical materials, polarization becomes nonlinear within the material. Here, we assume that the nonlinearity is local, allowing the dielectric constant of the nonlinear material to be expressed as a function of the electric field: $\varepsilon \left(r\right)=\varepsilon ({\left|E\left(r\right)\right|}^{2})$. In summary, metasurfaces with asymmetric structures and nonlinear materials can achieve nonreciprocal transmission.

The nonlinearity of the LMM is crucial for realizing the nonreciprocal transmission of metasurfaces, and the physical mechanism of nonlinearity needs to be explained in detail. The origin of relatively high optical nonlinearity, first predicted by Palmer [40] in the case of dielectric sphere aerosols, is attributed to the optical gradient force acting on the dielectric particles. This force changes the concentration of colloidal particles, thereby increasing the refractive index contrast in regions of higher light intensity. The pioneering work of Ashkin [41] established that optical gradient forces define the dynamics of light–matter interactions in colloidal suspensions. Light–matter interactions in colloidal systems can be significantly altered by modifying the polarizability of the particles, which depends on their refractive index relative to the background medium. Particles with a refractive index greater than that of the background medium exhibit positive polarizability. As the laser beam travels through this medium, radiation pressure attracts these particles to high-intensity regions. Conversely, particles with a refractive index lower than the background medium exhibit negative polarizability. Colloidal suspensions exhibit high nonlinearity. In the work of E. L. Falcao-Filho et al. [42], the nonlinearity of silver nanoparticle colloids in carbon disulfide was $10^{-16}(\frac{m^{2}}{V^{2}})$, which is six orders of magnitude higher than that of silica. The third-, fifth-, seventh-, and ninth-order magnetic susceptibilities for the optical nonlinearity of aqueous colloids containing silver nanoparticles (NPs) were also reported in their study [43], along with the relationship between the magnetic susceptibility and nanoparticle volume fraction. The LMM is assumed to be formed from a suspension of equally polarized NPs in a viscous liquid. The size of the NPs is believed to be much smaller than any other relevant scale in the theory, such as the wavelength of electromagnetic radiation. Additionally, the concentration of the NPs is assumed to be sufficiently high, and their number within the field heterogeneity scale is much greater than one. Here, the suspension can be regarded as a continuous medium, and its effective dielectric constant depends on the packing fraction of the NPs. The medium is assumed to be macroscopically isotropic, and its effective dielectric constant $\varepsilon_{eff}\;$is given as follows [44]:

(9) $$\varepsilon_{eff}=\varepsilon_{m}\left(1+\frac{3\beta v}{1-\beta v}\right)$$

with the local field factor β given by

(10) $$\beta =\frac{\varepsilon_{A}-\varepsilon_{m}}{\varepsilon_{A}+2\varepsilon_{m}}$$

By combining Equations (9) and (10), we obtain

(11) $$\varepsilon_{eff}=\varepsilon_{m}\left(1+3v\frac{\varepsilon_{A}-\varepsilon_{m}}{\varepsilon_{A}\left(1-v\right)+\varepsilon_{m}\left(2+v\right)}\right)$$

where $\varepsilon_{m}\;$and $\varepsilon_{A}$ represent the relative dielectric constants of the liquid medium and the NP material, respectively. The packing fraction of NPs is denoted as v. The effective third-order susceptibility $\chi_{eff}^{\left(3\right)}$ for small values of v is given in Ref. [43].

(12) $$\chi_{eff}^{\left(3\right)}=v\frac{\chi_{A}^{\left(3\right)}}{G^{2}{\left|G\right|}^{2}}+\frac{\chi_{m}^{\left(3\right)}\left(1-v\{1-0.4\left[4{\left|\beta \right|}^{2}\beta^{2}+3\left({\left|\beta \right|}^{2}\beta +\beta^{3}\right)+9\left({\left|\beta \right|}^{2}+\beta^{2}\right)\right]\}\right)}{{\left|1-\beta v\right|}^{2}{\left(1-\beta v\right)}^{2}}$$

with

(13) $$G=\left(1-\beta v\right)\frac{\varepsilon_{A}+2\varepsilon_{m}}{3\varepsilon_{m}}$$

where $\chi_{A}^{\left(3\right)}$ and $\chi_{m}^{\left(3\right)}$ represent the diagonal nonlinear susceptibilities of the NPs and the host medium, respectively. When the values of $\varepsilon_{A}$ and $\varepsilon_{m}$ are known, Equation (12) can be simplified as follows:

(14) $$\chi_{eff}^{\left(3\right)}≅v\left(a+ib\right)\chi_{A}^{\left(3\right)}+\chi_{m}^{\left(3\right)}$$

where $a+ib=\frac{1}{G^{2}{\left|G\right|}^{2}}$.

The nonlinear behavior of the colloid was determined by the local-field spatial variations, since the electric field induced by the laser beam at each NP differed from the average field in the colloid. The local fields were larger than the average, enhancing the observed optical response. Equation (14) shows that the nonlinearity of the LMM arises from the inherent nonlinearities of the metal NPs and liquid medium. Gold NPs exhibit strong third-order nonlinear optics due to their quantum confinement effect, surface effect, and surface plasmon polaritons that conduct electrons, and they have become the main contributors to nonlinear responses in LMMs. The dependence of $n_{2}\propto Re\chi_{eff}^{\left(3\right)}$ and $\alpha_{2}\propto Im\chi_{eff}^{\left(3\right)}$ on v can be described through the generalized Maxwell Garnett model introduced in Ref. [44]. Using Equation (14), and considering that $n=n_{0}+\left(n_{2}/2\right){\left|E\right|}^{2}$ and $\alpha =\alpha_{0}+\left(\alpha_{2}/2\right){\left|E\right|}^{2}$, we can obtain the second-order nonlinear refractive index coefficient $n_{2}$ from Equation (15), and the imaginary part $\alpha_{2}$ of the refractive index is given by Equation (16):

(15) $$n_{2}=\frac{3}{4n_{0}}\;Re\chi_{eff}^{\left(3\right)}=\frac{3}{4n_{0}}\left\{v\left[aRe\chi_{A}^{\left(3\right)}-bIm\chi_{A}^{\left(3\right)}\right]+Re\chi_{m}^{\left(3\right)}\right\}$$

(16) $$\alpha_{2}=\frac{3\omega }{2cn_{0}}\;Im\chi_{eff}^{\left(3\right)}=\frac{3\omega }{2cn_{0}}\left\{v\left[aIm\chi_{A}^{\left(3\right)}+bRe\chi_{A}^{\left(3\right)}\right]+Im\chi_{m}^{\left(3\right)}\right\}$$

where c is the speed of light, $n_{0}$ is the linear refractive index, and ω is the angular frequency. The possible contributions to the nonlinear response of the colloid arise from electronic and nuclear interactions within the liquid medium molecules and electronic transitions in the metallic NPs (including intraband transitions and surface plasmon excitations). The nonlinearity of the LMM is a linear function of the NP volume fraction and can be analyzed using the generalized Maxwell Garnett model, which allows for the evaluation of the nonlinear susceptibility of the NPs.

In this study, we used simulations to investigate optical nonreciprocal transmission based on the nonlinearity of the LMM. Additionally, we used the process described in Ref. [28] to rapidly obtain the value of the dielectric constant $\varepsilon_{eff}$ required by simulation. Ref. [28] assumes that dielectric colloidal particles interact through a hard-sphere potential. In the steady state, the colloidal particles satisfy the Maxwellian velocity distribution, derived from the phase space density in the canonical ensemble $\rho \sim exp\left(-E/k_{B}T\right)$. The pressure exerted by the colloidal particles can be determined using the equation of state, analogous to that of a hard-sphere gas:

(17) $$\frac{P_{e}}{k_{B}T\rho }=Z_{\left(v\right)}$$

where $P_{e}$ represents the pressure, $k_{B}$ is the Boltzmann constant, T is the temperature, $v=\rho /\rho_{0}$, ρ represents the colloidal particle density, and $Z_{\left(v\right)}$ represents the compressibility factor. For an ideal gas, Z=1, whereas for a hard-sphere gas, the Carnahan–Starling equation of state provides an accurate approximation of Z as $Z\approx (1+v+v^{2}-v^{3})/{\left(1-v\right)}^{3}$. This approximation remains valid up to the fluid–solid transition at v≈0.5 [45]. This phenomenological formula is consistent with exact perturbation theory calculations and molecular dynamics simulations.

Under a slowly varying external potential, such as one induced by an optical field, the particle velocity distribution follows a local Maxwellian distribution. Assuming that the density gradient ρ(r) is locally parallel to $\tilde{x}$, we analyzed a differential volume element dV=dxdS with the length dx and the normal plane dS. The pressure difference applied to the left and right surfaces ${dP}_{e}$ exerted a force on the colloidal particles $F_{int}$. This force was equivalent to the external force necessary to maintain the density gradient, $dP_{e}=-F_{int}/dS=-f_{int}\;\rho dV/dS=-f_{int}\;\rho dx$, where $f_{int}$ is the average force acting on a single particle. From Equation (17), we obtained $d(\rho Z)/dx=-f_{int}\;\rho \frac{1}{k_{B}T}$. The particle current density J is expressed as follows:

(18) $$J=\rho \mu \left(f_{ex}+f_{int}\right)=\rho \mu f_{ex}-D\nabla \left(\rho Z\right)$$

where μ represents the particle mobility, $f_{ex}$ represents the electromagnetic force, and $D=\mu k_{B}T$ represents the diffusion coefficient. In the ideal gas limit, the equation becomes Equation (19). The polarizability of a sphere is given by

(19) $$\alpha =3V_{0}\varepsilon_{0}\varepsilon_{m}(\frac{\varepsilon_{A}-\varepsilon_{m}}{\varepsilon_{A}+2\varepsilon_{m}})$$

where α is the particle polarizability, while $V_{0}$ represents the NPs’ volume. Seeking a steady state (J=0) in the presence of an optical field gradient ($f_{ex}=\frac{\alpha }{4}\nabla I$, where incident intensity $I={\left|E\right|}^{2}$), we obtain

(20) $$\rho \frac{\alpha }{4k_{B}T}\frac{dI}{dx}=\frac{d\left(\rho Z\right)}{dx}$$

This equation shows that a particle with positive polarizability experiences a force pulling it toward the beam. On the other hand, particles with negative polarizability experience a repelling force pointing outward, which can be solved analytically to give the dependence $I_{\left(v\right)}$:

(21) $$\frac{\alpha }{4k_{B}T}I_{\left(v\right)}=g\left(v\right)-g(v_{0})$$

where $g\left(v\right)=\frac{3-v}{{\left(1-v\right)}^{3}}+lnv$, and $v_{0}$ is the background packing fraction. For small v, this result is equivalent to the exponential dependence derived in Refs. [46,47].

(22) $$v=v_{0}exp\left(\frac{\alpha }{4k_{B}T}I_{\left(v\right)}\right)\approx v_{0}\left(1+\frac{\alpha }{4k_{B}T}{\left|E\right|}^{2}\right)$$

Therefore, by substituting Equation (22) into Equation (11), an approximate expression for the nonlinear dielectric constant of an LMM can be obtained as follows:

(23) $$\varepsilon_{eff}\left({\left|E\right|}^{2}\right)\approx \varepsilon_{m}\left(1+3v_{0}\frac{\varepsilon_{A}-\varepsilon_{m}}{\varepsilon_{A}+{2\varepsilon }_{m}}\left(1+\frac{\alpha }{4k_{B}T}{\left|E\right|}^{2}\right)\right)$$

The simplistic model considered here cannot be applied directly to the real world, because the electromagnetic and pressure gradient forces cannot be viewed in reality, nonlinearity cannot be treated as local, and the overall scattering problem must be solved self-consistently. However, these calculations were performed only numerically. Therefore, this method could be used when conducting research through simulations in this study. Meanwhile, the structure proposed in this paper also has feasible manufacturing methods in practice. For example, we can prepare solid nanostructures by using standard nanofabrication techniques. Silicon and silicon oxide layers can be stored by chemical vapor deposition. Electron-beam lithography technology can visualize grating structures under appropriate resists and masks. Finally, after the structure is completed through ion etching, the mask should be removed. The LMM used in this study also has feasible manufacturing methods. The required LMM was synthesized by the methods outlined in [42,43,48], and the optical isolation performance of the metasurface was evaluated by the method described in [49].

As can be ascertained from the discussion of Equations (17)–(23), light–matter interactions in colloidal systems can be significantly altered by changing the particles’ polarizabilities, which depend on the refractive indices of the particle and the background medium. It was first discussed in detail by El-Ganainy [47] that, in both cases (of positive and negative polarizability), the nonlinearity is of the self-focusing type. This rather unexpected conclusion can be understood as follows: In the event that the particles have a higher refractive index than the background medium, the polarizability of each particle is positive and, thus, the particles are attracted toward the high-intensity region, i.e., to the center of the beam, thus elevating the effective refractive index of the system. Obviously, this will increase the nonlinear scattering losses as well. On the other hand, particles with a lower refractive index than that of the background medium—and, hence, a negative polarizability—will be repelled away from the center of the beam, again effectively increasing the refractive index at the center. The important difference, however, is that in the case of particles with negative polarizability, the nonlinear losses decrease at the beam’s center due to the reduction in the particle concentration, thus increasing the transparency of the system. This difference in the type of the exponential optical nonlinearity in these two cases was shown to have a significant impact on the dynamics of spatial soliton propagation in such media.

## 3. Results and Discussion

Full-wave simulations were conducted using COMSOL Multiphysics 6.0 with an RF frequency-domain solver. The metasurface was modeled as a periodic structure with a primitive unit cell incorporating periodic boundary conditions. The unit cell contained a metasurface consisting of three layers (Figure 1). The dielectric properties of silicon and silicon dioxide were obtained from a refractive index database [50]. The LMM was modeled as an aqueous suspension of 10 nm gold NPs, with the dielectric properties of gold NPs and water obtained from the refractive index database [50]. The effective nonlinear permittivity of the LMM was computed using Equation (23). A plane EW of a given frequency was incident on the metasurface from both the forward and backward directions. Finally, the transmission coefficients were calculated.

**Figure 2:**
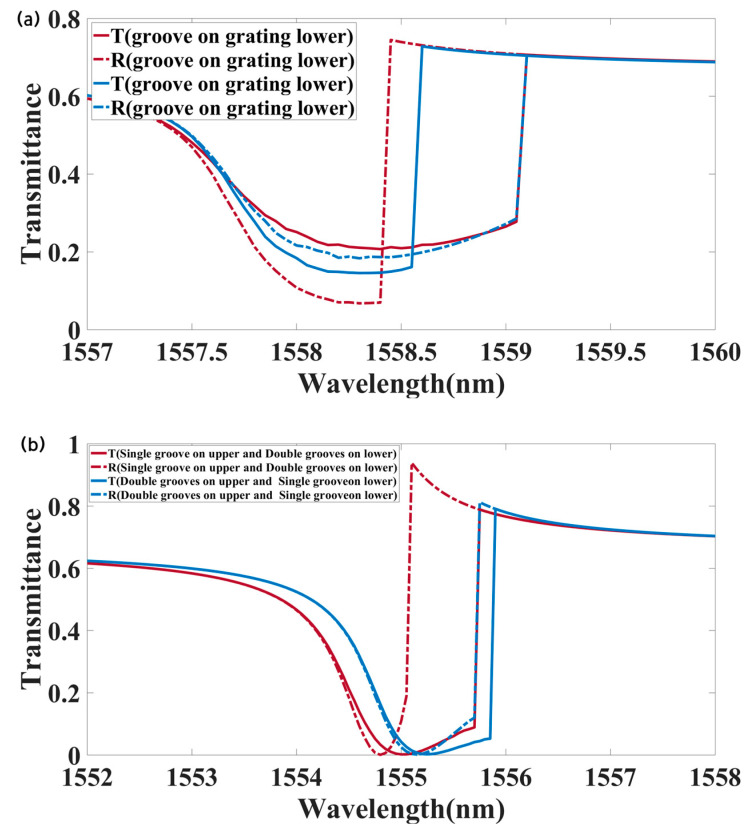
Wavelength-dependent transmission coefficients for forward and backward propagation: (**a**) single-sided grating; (**b**) double-sided grating.

We analyzed the transmission characteristics of metasurfaces with different structural configurations to achieve effective optical isolation, as shown in Figure 2. First, grooves were etched into the upper surface of the silicon layer of the metasurface, forming a single-sided grating structure (called Str1), where the parameters were set as follows: P = 600 nm, H = 200 nm, l = 180 nm, w = 80 nm, and h = 10 nm. The forward and backward transmissions of the metasurface are represented by the blue line in Figure 2a, where the solid line denotes forward transmission and the dashed line denotes backward transmission. The incident light intensity was 18 kW/cm^2^, and the background packing fraction v_0_ was 0.004. With the structural parameters unchanged, the groove was relocated to the lower surface to form a new structure (called Str2), and its transmission response is represented by the red line in Figure 2a. As illustrated in Figure 2a, a grating with a groove on the upper surface enabled forward optical isolation, while a groove on the lower surface facilitated backward optical isolation. The two periodic sections of the upper surface grating were combined into a single period, resulting in a new grating with P = 1200 nm, while all other parameters remained unchanged. A groove was introduced at the center of the lower surface of the silicon layer in the merged grating, forming a double-sided grating (called Str3). The transmission characteristics of the new structure are represented by the blue line in Figure 2b, with the solid line indicating forward transmission and the dashed line indicating backward transmission. Similarly, a double-sided grating with a single groove on the upper surface and a double groove on the lower surface can be designed (called Str4). The transmission response of this configuration is depicted by the red line in Figure 2b. Table 1 presents the performance under four different structures. Notably, the highest transmission contrast was achieved for the double-sided grating structure with a single groove on the upper surface and a double groove on the lower surface.

**Figure 3:**
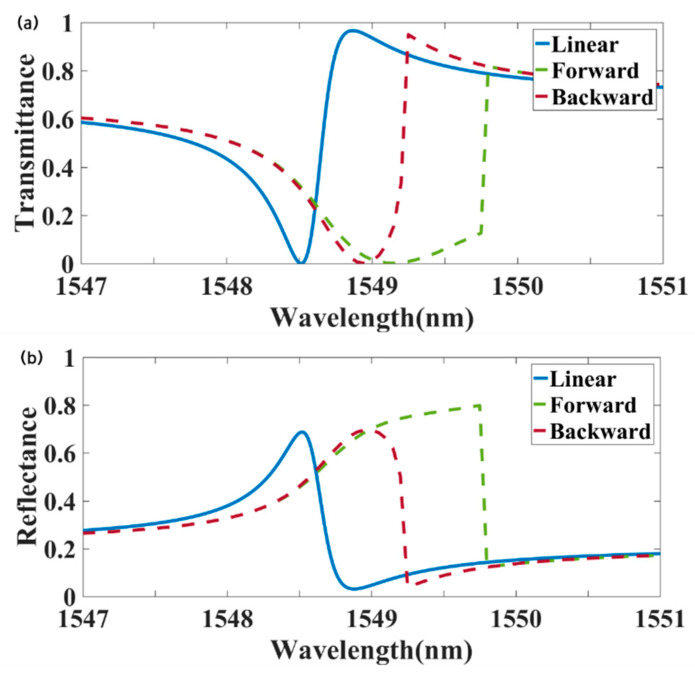

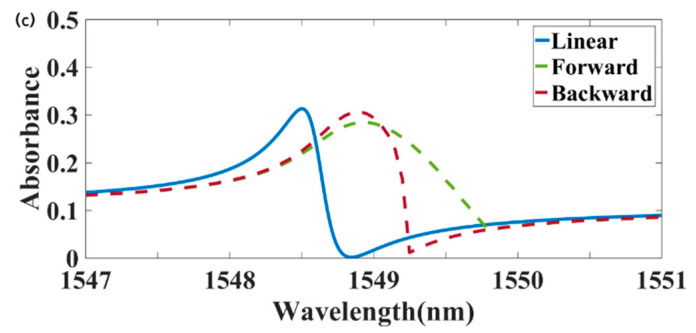
Spectral response of the metasurface: (**a**) transmittance; (**b**) reflectivity; (**c**) absorptivity.

Figure 3 presents the transmission, reflection, and absorption spectra of the metasurface under a radiation power of 18 kW/cm^2^, with a background packing fraction, v_0_ = 0.004, and the following structural parameters: P = 1192 nm, H = 200 nm, l = 181 nm, w = 80 nm, and h = 10 nm. The solid line represents the linear transmittance of forward-incident light at an intensity of 1 W/m^2^. At a wavelength of 1549.2 nm, the forward transmittance was 0.004, while the backward transmittance was 0.964, yielding a transmittance contrast of 0.96, and demonstrating strong nonreciprocity. The proposed design achieved a transmittance contrast exceeding 70% over a wavelength bandwidth of 0.5 nm. This strong nonreciprocal transmission capability has potential applications in optical communications, light detection and ranging (LiDAR), and related technologies. For example, nonreciprocal devices, such as optical isolators and circulators, are vital LiDAR systems enabling unidirectional light propagation, protecting the pulsed laser source from the reflected pulse stream, and ensuring system stability. Recently, a nonreciprocal pulse router for chip-based LiDAR was demonstrated, achieving a range of up to 60 m with a nonreciprocal transmission bandwidth of 0.08 nm [51]. These findings highlight the strong application potential of metasurface-based optical nonreciprocal devices.

**Figure 4:**
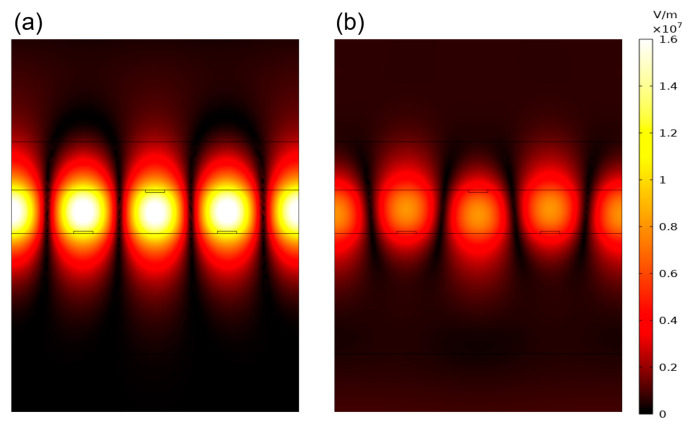
Electric field intensity distributions at 1549.2 nm under an incident light intensity of 18 kW/cm^2^: (**a**) forward incidence; (**b**) backward incidence.

Figure 4a,b illustrate the forward and backward electric field intensity distributions at a wavelength of 1549.2 nm under an incident light intensity of 18 kw/cm^2^. The electric field intensity generated by the forward-incident light was significantly greater than that of the backward-incident light.

**Figure 5:**
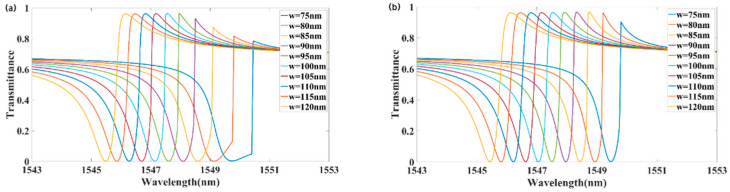
Effect of the groove width w on the transmission spectra: (**a**) forward incidence; (**b**) backward incidence.

We optimized the metasurface’s performance by adjusting its geometric parameters to enhance nonreciprocal transmission. First, we analyzed how the structural parameters influence nonreciprocity. Figure 5a illustrates the transmission for forward incidence. With all other parameters and the incident intensity unchanged, decreasing the groove width w from 120 to 75 nm shifted the transmission peak from 1546.1 to 1550.5 nm, resulting in a redshift of approximately 4.4 nm. As w decreased, the stepwise shift of the transmission peak increased, while its magnitude gradually decreased. Figure 5b shows the transmission of backward incidence. Decreasing w from 120 to 75 nm shifted the transmission peak from 1546.1 to 1549.8 nm, resulting in a redshift of approximately 3.7 nm. As w decreased from 80 to 75 nm, the peak reverse transmission decreased significantly. As shown in Figure 5, at w = 80 nm, the difference between forward and backward transmittance was maximized, while the operational bandwidth of nonreciprocal transmission increased as w decreased.

**Figure 6:**
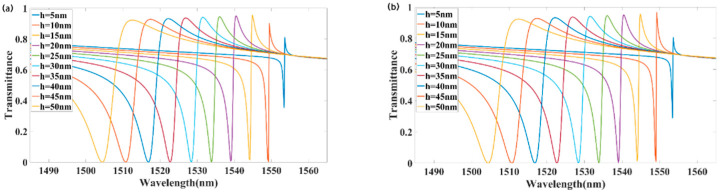
Effect of the groove depth h on the transmission spectra: (**a**) forward incidence; (**b**) backward incidence.

Second, we analyzed the effect of varying the grating groove depth h on transmission. Figure 6a shows the forward-incident transmission spectra for different values of h. Decreasing the groove depth h from 50 to 5 nm shifted the transmission peak from 1512.4 to 1553.6 nm, resulting in a redshift of approximately 41.2 nm. At h = 15 nm, the transmission peak reached its maximum value. As h decreased, the transmission peak also decreased. Figure 6b shows the transmission for backward incidence. Reducing h from 50 to 10 nm shifted the transmission peak from 1512.4 to 1549.2 nm, resulting in a redshift of approximately 36.8 nm. When h was reduced to 5 nm, the backward transmission nearly coincided with the forward transmission line. As shown in Figure 6, the device exhibited optimal optical isolation at h = 10 nm.

**Figure 7:**
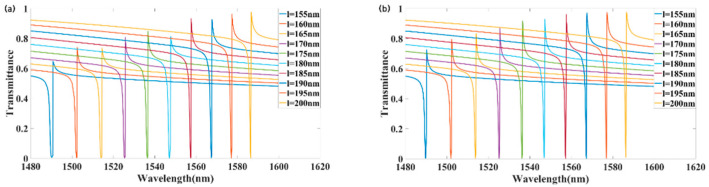
Effect of silicon layer thickness l on transmission spectra: (**a**) forward incidence; (**b**) backward incidence.

We also adjusted the silicon layer thickness l to examine its effect on metasurface transmittance. Figure 7a,b illustrate the forward and reverse transmittances for various values of l. As l increased, the maximum transmissivity of the metasurface resonance increased correspondingly. As l increased from 155 to 200 nm, the peak forward transmittance increased from 0.648 to 0.976, while the peak backward transmittance increased from 0.726 to 0.948. At l = 175 nm, the reverse transmittance first exceeded 0.9, and after fine-tuning l, it reached 0.964 at l = 181 nm.

**Figure 8:**
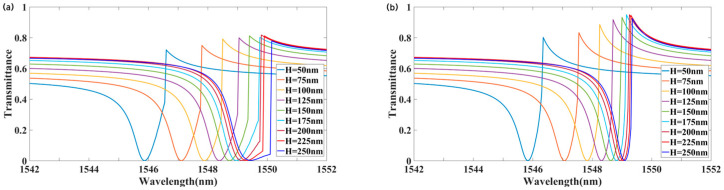
Effect of LMM thickness H on transmission spectra: (**a**) forward incidence; (**b**) backward incidence.

Additionally, the thickness of the LMM was varied to analyze its impact on transmission. Figure 8 illustrates the transmission for different values of H, showing a redshift in the resonance wavelength as H increases. Comparing Figure 8a,b, for H > 200 nm, the transmittance difference between forward and backward incidence exceeded 0.8. At H = 250 nm, the peak backward transmittance was 0.925 at 1549.3 nm, while the forward transmittance was 0.006, yielding a transmittance contrast of approximately 0.92. The nonreciprocal transmission bandwidth reached 0.9 nm. As shown in Figure 6, for H > 200 nm, both the forward and backward transmission peaks decreased significantly, and the transmittance contrast decreased as H increased. In summary, increasing H extends the operational bandwidth of nonreciprocal transmission but reduces optical isolation.

**Figure 9:**
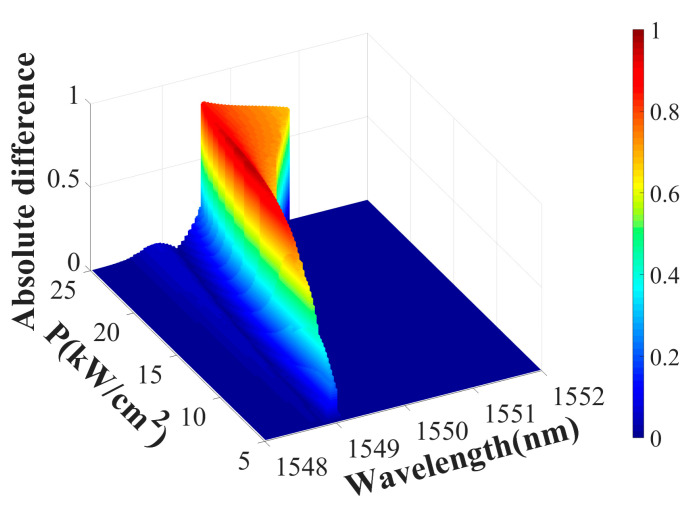
Absolute difference between forward and backward transmission as a function of the radiation wavelength and power.

**Figure 10:**
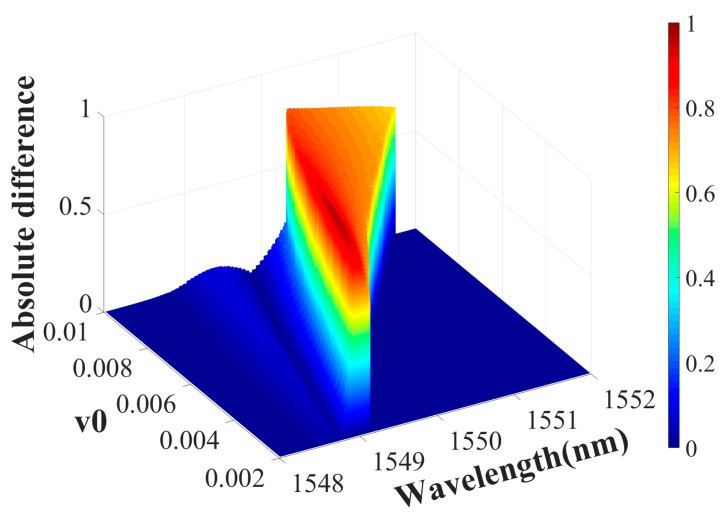
Absolute difference between forward and backward transmission as a function of radiation wavelength and background packing fraction.

The metasurface system under consideration exhibits several notable characteristics. First, it facilitates strong optical nonreciprocity in an ultrathin layer. Notably, the simulated metasurface thickness was below 1 µm, smaller than the incident wavelength. Despite its ultrathin profile, the system exhibited a transmission coefficient contrast of up to 0.96 between opposite propagation directions. The metasurface can be optimized for specific wavelengths by tuning its structural parameters, demonstrating high adaptability. Second, incorporating an LMM significantly lowers the radiation power needed to induce nonlinearity. Figure 9 shows the absolute difference between the forward and backward transmittance of the metasurface under different incident light intensities. As the incident power increases, the dielectric constant distribution of the LMM shows greater differences in different cases of forward and backward incidence, increasing the working bandwidth of nonreciprocal transmission. The average dielectric constant of the LMM increases with the increase in power, reducing the maximum value of the absolute difference. Notably, optical isolation in the proposed system is achieved at a power density of 7 kW/cm^2^ (Figure 9), whereas in natural materials, inducing sufficient nonlinear effects typically requires power densities reaching GW/cm^2^. This significant power reduction stems from the unique properties of the LMM. Finally, varying the NP volume fraction in the LMM modulates the transmission characteristics. In Figure 10, as the background packing fraction v_0_ increases, it can be known from Equations (13) and (14) that the nonlinearity of the LMM also increases under the same incident light intensity. Therefore, the change in the absolute difference of the forward and backward transmittance is similar to that in Figure 9, with the bandwidth increasing and the maximum value decreasing. Thus, the LMM exhibits dual regulation properties.

However, the proposed design has certain limitations. First, effective optical isolation is confined to a narrow frequency range. This is because the nonreciprocal transmission depends on the excitation of the eigenmode of the grating, which is inherently constrained by its resonance width. Moreover, the actual nonreciprocal transmission bandwidth is narrower than the resonance width of the grating, because sufficient asymmetry in the excited mode, relative to the propagation direction, arises only within a limited frequency range. Consequently, the proposed metasurface is unsuitable for broadband applications. Second, the LMM’s nonlinearity is significantly lower than the characteristic timescale of electromagnetic radiation, as it arises from the physical displacement of particles in a liquid, which requires time. Therefore, the proposed system is unsuitable for applications requiring fast switching. Finally, nonlinear, nonreciprocal devices have intrinsic limitations. For instance, such devices cannot function with multiple simultaneous excitation signals [52]. Additionally, the performance of LMM-based devices is temperature-sensitive. As the temperature increases, particle motion intensifies, decreasing the particle density, packing fraction v_0_, refractive index, and third-order magnetic susceptibility. However, the optical properties of the LMM exhibit significant variations in supercooled and superheated environments, necessitating further investigation.

## 4. Conclusions

This study introduces a nonreciprocal metasurface design incorporating an LMM with high nonlinearity. The proposed metasurface, comprising a 2D grating immersed in an LMM, demonstrates strong nonreciprocity in its transmission properties. The metasurface’s Si grating structure enhances the resonance intensity and achieves the differential distribution of the electric field when different light is incident in the forward and backward directions. The nonlinearity of the LMM makes its dielectric constant related to the electric field intensity. The combination of the two structures breaks the Rayleigh–Carson reciprocity theorem and achieves nonreciprocal light propagation. Theoretical analysis and numerical simulations at a light intensity of 18 kW/cm^2^ and wavelength of 1549.2 nm revealed a strong nonreciprocity effect, with a forward transmittance of only 0.004, reverse transmittance of 0.964, and transmittance contrast of 0.96. This design holds promise for applications such as optical isolators and asymmetric power limiters, where nonreciprocal electromagnetic response is crucial. Leveraging LMMs enables extremely high nonlinearity, significantly reducing the radiation power needed to induce nonlinear effects, including nonreciprocal transmission. Future research should explore further optimizations and alternative metasurface geometries, or use materials with better performance (for example, it is possible to replace Si with a material with smaller refractive index) and the potential for integration with other functional elements to improve the overall performance.

## Figures and Tables

**Table 1:** Performance comparison of four different structures.

Structure	Isolation Direction	Transmittance Difference	Bandwidth (nm)
Str1	Forward	0.530	0.5
Str2	Backward	0.532	0.6
Str3	Backward	0.768	0.15
Str4	Backward	0.934	0.65

## Data Availability

The original contributions presented in this study are included in the article material. Further inquiries can be directed to the corresponding author.

## Abbreviations

The following abbreviations are used in this manuscript:

LMM	Liquid metamaterial
SCMPC	Superconducting ceramic superstructure photonic crystal
EW	Electromagnetic wave
2D	Two-dimensional
NP	Nanoparticle
LiDAR	Light detection and ranging
